# Scheduling IT Staff at a Bank: A Mathematical Programming Approach

**DOI:** 10.1155/2014/768374

**Published:** 2014-03-17

**Authors:** M. Labidi, M. Mrad, A. Gharbi, M. A. Louly

**Affiliations:** Department of Industrial Engineering, College of Engineering, King Saud University, P.O. Box 800, Riyadh 11421, Saudi Arabia

## Abstract

We address a real-world optimization problem: the scheduling of a Bank Information Technologies (IT) staff. This problem can be defined as the process of constructing optimized work schedules for staff. In a general sense, it requires the allocation of suitably qualified staff to specific shifts to meet the demands for services of an organization while observing workplace regulations and attempting to satisfy individual work preferences. A monthly shift schedule is prepared to determine the shift duties of each staff considering shift coverage requirements, seniority-based workload rules, and staff work preferences. Due to the large number of conflicting constraints, a multiobjective programming model has been proposed to automate the schedule generation process. The suggested mathematical model has been implemented using Lingo software. The results indicate that high quality solutions can be obtained within a few seconds compared to the manually prepared schedules.

## 1. Introduction

Scheduling of staff or personnel is applicable in a wide variety of areas and service delivery fields such as scheduling of medical personnel in hospitals, scheduling of airline and hotel personnel, and scheduling of patrol officers. A good schedule balances the needs of employees or customers and the tasks involved, and schedules can be created for various periods of time such as days, weeks, or months. Depending on the complexity of the schedule, some organizations can use scheduling software such as spread sheets software to complete the task. One thing to note regarding software is that it can be expensive and can also be catastrophic for large organizations if poorly managed.

Some of the most common challenges with scheduling arise due to scheduling conflicts or poorly arranged schedules. Scheduling conflicts arise, for example, when an employee is scheduled to work at two locations at the same time. This is usually caused by changes made to the schedule by different people or lack of verification of the impact of the change on the rest of the schedule. Poorly arranged schedules include features such as lack of breaks between shifts and double shifts. This leads to poor production due to low staff morale and overwork as well as increasing the risk of accidents or mistakes due to fatigue of the employees.

In their simplest form, these problems involve only the assignment of days off, for example, in some of the less complex settings for the scheduling of nurses. A typical problem of this form requires the scheduler to give appropriate days off to each of a number of employees who work standard shifts while assuring that the required numbers of employees are on duty throughout the day and week. Effective staff scheduling consists of allocating the required workload to staff subject to a number of constraints. The main objectives of staff scheduling are to efficiently utilize resources, produce a schedule with a balanced workload, and satisfy individual preferences as much as possible.

There are various scheduling methods available and are discussed briefly below. The chosen scheduling method should conform to the department workload pattern to enable creation of effective and efficient employee schedules. Some scheduling models include fixed schedule method, rotation schedule method, pattern schedule method, coverage schedule method, assignment schedule method, and team schedule method. The fixed schedule method is mainly used where the workload is predictable; that is, the work is consistent and does not require a lot of flexibility. The rotation schedule method is used to ensure a balance between shifts; for example, the employees share the burden to be covered in 24 hrs in shifts such as morning shifts and evening shifts. Pattern schedule method sets the shift rotation by day of the week and is mainly applied where the work days usually change cyclically from one schedule period to another. An example of the pattern schedule method is where every employee is off every 2nd weekend. The coverage schedule method is used where the pattern of the workload is not consistent and a lot of flexibility is required. In this method, schedules are set according to the prevailing pattern in the workload. Assignment schedule method combines the aspect of time when one will be working and the assignment to be carried out; for example, at different times an employee will be in different departments. In the team schedule method, the employees are scheduled as a group; that is, there is one schedule for a group of workers following the same pattern or completing the same tasks.

The remainder of this paper is organized as follows. In Section 2, we begin by giving some literature review. Next, we propose a multiobjective model with the description of notation and variables, constraints, and objective function. In Section 3, we report the results of a computational study of the proposed approach. Finally, in the last section we provide some concluding remarks and directions for future investigations.

## 2. Literature Review

Creating a staff duty roster is very challenging and also time consuming. Although there is no polynomial known mathematical algorithm that guarantees an optimal solution in a reasonable computation time, it is still possible to generate duty rosters which are better than those produced by a human planner. Scheduling problems occur mostly when events are competing for the same attention, that is, conflicting constraints. The occurrence of conflicts can be stressful and also detrimental to productivity so it is very vital for managers to focus on prevention of these problems and, when they occur, work on minimizing the impact of the disruption. Two common ways to prevent scheduling conflicts are to review the full schedule during and after making changes and also have one person in charge of the schedule. The first method helps to ensure no new conflicts are generated or conflicts are detected early while the second reduces the risk of mistakes due to many people working on the same schedule. In this section, we discuss the approaches taken by several authors to find a solution to generate an effective schedule.

In [1], the authors proposed a review of applications used for staff scheduling and rostering problems. They have presented specific applications areas, models, algorithms, and methods used for solving them. In [2], a simulated annealing heuristic for use in a continuously operating scheduling environment is presented. Their research is designed to minimize the number of employees required to satisfy forecast demand. From the results, in terms of solution quality, the simulated annealing based method dominates other heuristics and it also exhibits rapid convergence to a low-cost solution.

One example of an NP-hard combinatorial problem is nurse rostering, and addressing scheduling problems can be done by using constraint programming and linear programming techniques. However, we note that, depending on the scenario, heuristics may also be applied for a feasible solution. For example, in [3], heuristics were necessary to address the issue of nurse rostering in Belgian hospitals. This approach was necessary due to the overconstrained schedules, the need to reduce the time to generate a schedule, and the fact that the cyclic three-shift schedule could not meet the hospital requirements. The Plane algorithm presented is used to determine which nurse can perform particular duties (according to qualification) and also when there are insufficient staff. The Tabu search method which is a local search method used for mathematical optimization for solving combinatorial optimization problems is used in the Plane algorithm. The two algorithms used by Plane are Tabu search and diversification and Tabu search and greedy shuffling. These algorithms are aimed at providing reliable solutions in a short time and providing results that are better from the human point of view, respectively. Results show that better qualities of rosters were provided and the users were more satisfied with the results. While addressing the limitations of the cyclic scheduling for nurses in Belgium, authors in [4] investigate the benefit of integrating nurse-specific characteristics into the cyclic scheduling approach. They analyze the extent to which the characteristics should be incorporated and this is compared to the cyclical and flexible acyclical scheduling approaches.

Genetic algorithms are search heuristics used to generate useful solutions as well as optimize and search problems. When implementing genetic algorithms for solving scheduling problems, however, there is a challenge handling the conflict between constraints and objectives which are prevalent in scheduling tasks. One approach to handling this challenge is to use problem specific knowledge, and in [5] genetic algorithms were used to address a nurse scheduling problem in a major UK hospital. In [5], the research investigates the effect of three types of problem specific knowledge on the scheduling challenge. The results show that the solution provided is fast, robust, and flexible.

The authors in [6] also showed how the column generation technique can easily be extended to integrate both nurse and surgery schedules to provide cost savings and reduce schedule generation times. In [7], the nurse rostering problem was partitioned into subproblems and each was solved sequentially using Integer Mathematical Programming (IMP). IMP is a mathematical optimization program which restricts variables to integers. In addition to local optimization techniques, their approach uses two phases where the first phase determines the workload for each nurse for each day of the week and the second phase allocates the daily shifts. Also, Mixed Integer Programing (MIP) is used in [8] to assign technicians to tasks requiring multilevel skill requirements by repeatedly applying a flexible matching model. This model selects tasks to be completed then forms groups of technicians assigned to a combination of tasks. The MIP model performs very well in the case of rare skills and is capable of revising technician-task allocations. Furthermore, a mathematical model for staff scheduling in a telecommunications center is proposed in [9] to replace the manually generated weekly schedule which was generating inefficient and unfair schedules. A zero-one linear goal programming model with the work patterns mathematically formulated as a set of soft constraints was used. The results show the optimal scheduling cycle and also significant increase in efficiency and staff satisfaction.

In [10], the authors focus on scheduling crew and aircrafts while considering possible disruption. The goals in this case are to reduce slack between flights and reserve resources more intelligently or efficiently. They research into methods of evaluating robust scheduling approaches for aircraft and crew and develop stability indicators based on delay propagation.

Branch and price methods are used for solving integer linear programming and mixed integer linear programming problems which include many variables. The branch and price methods have been applied to scheduling of train drivers in [11]. Their approach looks at minimizing the number of duties and maximizing the schedule for outside disruptions. A duty in this case consists of a sequence of trips carried out on a given day by a train driver. An implicit column generation solution approach is used where a dynamic programming algorithm for the pricing-out of columns forms the basis of the heuristic brand and price algorithm. Also, [12] uses the brand and price models to solve workforce scheduling problems and propose an integer multicommodity network flow formulation to solve the problem.

## 3. Problem Statement

This paper addresses scheduling constraints encountered by bank IT staff. The problem can be described as finding a schedule that both respects the constraints of the staff and fulfils the objectives of the organization. Currently, there is a manual process for producing the monthly schedules using excel sheets; however, there are scenarios where the generated schedule violates some constraints. In order to deal with this issue, an automated scheduling system which will take into account the numerous constraints and goals is required for supervisors and operators in the IT Department. The next paragraphs present the major constraints and data related to the research.

There are four types of shifts (1 shift = 8 hours):

- morning shift (M): 06:00–14:00,
- evening shift (E): 14:00–22:00,
- night shift (N): 22:00–06:00,
- external shift (S): 08:00–16:00.

The department rules or constraints to be taken into account while generating the schedule for IT staff can be summarized as follows.

1. There are 9 operators and 5 supervisors who work morning, evening, and night in 8-hour shifts to provide a service that operates 24 hours a day and 7 days a week.
2. The morning and evening shifts should include three employees (i.e., one supervisor and two operators).
3. Each employee is required to work an average of 40 hours per week.
4. The employee should not work on day shift after night shift.
5. Each employee must have at most one shift per day.
6. Overtime is estimated by the number of hours that exceed 40 hours per week.
7. Only operator staff can work at an external/outside branch.
8. Operator staff that leaves to work at an external/outside branch should have 2 days off on Thursday and Friday.
9. The preferences of day off of each employee are taken into consideration.

## 4. Model Formulation

The staff scheduling problem has been formulated as a multiobjective programming model. Considering the fact that some of the constraints might be conflicting, however, the model will try to reduce these violations by minimizing the number of overtimes and maximizing the preferences of the staff.

### 4.1. Notation

The following notations are used to specify the model.

*N*_*jkl*_: the requirement of staff of category *j* in shift *k* in day *l*,
*n*_*j*_: the number of employees of category *j*,
*S*_*w*_: the set of days of week *w*,
*S*_off_(*i*,*j*)__: the preferable set of days off of employee *i* of category *j*,
AL_*ij*_: the set of annual leave days of employee *i* of category *j*,
*s*_1*w*_: the first 5 days of each week *w*,
*s*_2*w*_: the 2 last days of each week (Thursday and Friday).

### 4.2. Decision Variables

The used decision variables can be described as follows:

(1) $$\begin{array}{c} x_{ijkl}=\{\begin{array}{cc} 1, & \text{if}\;\text{employee}\;\;i\;\;\text{of}\;\;\text{category}\;j\;\\ & \text{is}\;\text{working}\;\text{in}\;\text{shift}\;\;k\;\;\mathrm{on}\;\text{day}\;\;l\\ 0, & \text{otherwise}. \end{array} \end{array}$$

 
*D*_*ijw*_: the number of overtime shifts of employee *i* of category *j* in week *w*,
 
*R*_*ij*_: the number of days where the employee *i* of category *j* worked and normally he does not want to work,
  (2) $$\begin{array}{c} y_{iw}=\{\begin{array}{cc} 1, & \text{if}\;\text{employee}\;\;i\;\;\text{is}\;\text{working}\;\text{external}\\ & \text{shift}\;\text{during}\;\text{the}\;\text{week}\;\;w\\ 0, & \text{otherwise}. \end{array} \end{array}$$

### 4.3. Model Constraints

The department rules for scheduling have been modelled into constraints and presented in this section.

(1) Each employee should work at most one shift per day:
  (3) $$\begin{array}{c} \overset{4}{\underset{k=1}{\sum }}x_{ijkl}\leq 1\;\;\begin{array}{c} \forall i=1\cdots n_{j}\\ \forall j=1\cdots 2\\ \forall l=1\cdots 28.\; \end{array} \end{array}$$
(2) Set the staff requirement in each shift:
  (4) $$\begin{array}{c} \overset{n_{j}}{\underset{i=1}{\sum }}x_{ijkl}=N_{jkl}\;\begin{array}{c} \forall j=1\cdots 2\\ \forall k=1\cdots 4\\ \forall l=1\cdots 28. \end{array} \end{array}$$
(3) Each employee should not work in annual leave days:
  (5) $$\begin{array}{c} \overset{4}{\underset{k=1}{\sum }}\;\overset{}{\underset{l\in \mathrm{AL}(i,j)}{\sum }}x_{ijkl}=0\;\begin{array}{c} \forall i=1\cdots n_{j}\\ \forall j=1\cdots 2. \end{array} \end{array}$$
(4) The employee should not work on day shift after night shift:
  (6) $$\begin{array}{c} x_{ij3l}+x_{ij1(l+1)}\leq 1\;\begin{array}{c} \forall i=1\cdots n_{j}\\ \forall j=1\cdots 2\\ \forall l=1\cdots 28. \end{array} \end{array}$$
(5) Overtimes will be considered starting from the sixth shift during each week *w*:
  (7) $$\begin{array}{c} \overset{3}{\underset{k=1}{\sum }}\;\overset{}{\underset{l\in s_{w}}{\sum }}x_{ijkl}-d_{ijw}\leq 5\;\begin{array}{c} \forall w=1\cdots 4\\ \forall i=1\cdots n_{j}\\ \forall j=1\cdots 2. \end{array} \end{array}$$
(6) Overtimes should not exceed two shifts per week:
  (8) $$\begin{array}{c} d_{ijw}\leq 2\;\begin{array}{c} \forall w=1\cdots 4\\ \forall i=1\cdots n_{j}\\ \forall j=1\cdots 2. \end{array} \end{array}$$
(7) Counting the number of working shifts during the preferable off days:
  (9) $$\begin{array}{c} \overset{4}{\underset{k=1}{\sum }}\;\overset{}{\underset{l\in S_{\text{of}{\text{f}}_{\left(i,j\right)}}}{\sum }}x_{ijkl}=R_{\left(i,j\right)}\;\begin{array}{c} \forall i=1\cdots n_{j}\\ \forall j=1\cdots 2. \end{array} \end{array}$$
(8) The operator who leaves to work at an external branch should take two days off per week (Thursday and Friday):
  (10) $$\begin{array}{c} \overset{}{\underset{L\in s_{1w}}{\sum }}x_{i24l}=5y_{iw}\;\begin{array}{c} \forall i=1\cdots n_{2}\\ w=1\cdots 4; \end{array}\\ \overset{4}{\underset{k=1}{\sum }}\;\overset{}{\underset{l\in s_{2w}}{\sum }}x_{i2kl}\leq 2\left(1-y_{iw}\right)\;\begin{array}{c} \forall i=1\cdots n_{2}\\ w=1\cdots 4. \end{array} \end{array}$$

### 4.4. Objective Function

The objectives are to minimize the overtimes and maximise the satisfaction of employees in terms of desired days off. These objectives are weighted according to their priority such that (*β*1 > *β*2)

(11) $$\begin{array}{c} \mathrm{Min}Z=\;\beta 1\overset{n_{j}}{\underset{i=1}{\sum }}\;\overset{2}{\underset{j=1}{\sum }}\;\overset{4}{\underset{w=1}{\sum }}d_{ijw}+\beta_{2}\overset{n_{j}}{\underset{i=1}{\sum }}\;\overset{2}{\underset{j=1}{\sum }}R_{ij}. \end{array}$$

The highest importance level (*β*1) is given to minimize the overtime, whereas the second highest priority level (*β*2) is given to satisfaction of desired employee preferences. Since our model involves determining the weights of the two objectives, therefore, for our problem we use the two levels; level 2 represents the major objective of the problem: to minimise the overtime. Level 1 represents the degree of satisfaction of employee preferences. The weights *βi*  (*i* = 1,2) will be equal to the grade of the level (*β*1 = 2 and *β*2 = 1).

## 5. Results

The following section presents the results of the research. Shift notations are summarized in Table 1. The results for the supervisor staff are shown in Table 2 (old solution) and Table 3 (new solution). These results compare the generated schedules of the working shift of each supervisor per day over a 4-week period. The results for the operator staff are shown in Table 4 (old solution) and Table 5 (new solution). These results compare the generated schedules of the working shift of each operator per day over a 4-week period.

### 5.1. Supervisors Shift

From comparing the generated schedules using the old and new methods, there are several things to be noted. First of all, the old solution gave total overtime of 8 shifts while the new solution has total overtime of only 4 shifts. This represents a 50% reduction in overtime costs. In addition, the old solution showed two supervisors (Supervisors 3 and 4) with higher amounts of overtime compared to the rest which was an indication of unfair allocation of work. In the new solution, however, the overtime is more distributed indicating that the work has been more fairly allocated. It should also be noted that in the new solution, on two occasions, the overtime was taken during the vacation period. This is preferable to taking extra hours on a normal working day especially if this overtime occurs at the beginning or end of the supervisor vacation. In the old solution, supervisors could be seen working for more than five days without a day off while this is not visible in the new solution. Finally, the new solution has implemented the supervisors' shift preferences and this can be seen through Supervisor 2 who is mostly on the morning shift and Supervisor 4 who is mostly on the evening shift. From analysis of the results, we can conclude that the initial objectives of reducing overtime and catering for supervisor preferences have been achieved.

### 5.2. Operators Shift

From comparing the generated schedules using the old and new methods, there are several things to be noted for the operators' shifts. First of all, the old solution gave total overtime of 16 shifts while the new solution has total overtime of 15 shifts. This represents a 6.25% reduction in overtime costs. The smaller reduction in overtime when compared to the supervisor case can be explained as due to the fact that there is an additional shift for operators and also more operators are required per shift compared to supervisors. When we compare the old and new solutions, there is a slightly better distribution of overtime; for example, in the old solution there are 3 staff with 2 shifts of overtime and 3 staff with 1 shift of overtime whereas in the new solution there are 2 staff with 2 shifts of overtime and 4 staff with 1 shift of overtime each. One limitation to note however is that with both the old and new solutions there are still cases of high amounts of overtime, for example, 3 and 4 shifts of overtime. This indicates that there is still some unfair allocation of work in some cases. Finally, we also note that the staffs' preferences have been catered for in the new solution (see, e.g., Operator 9 who is majorly on the evening shift). We also note that all operators have more regular days off when compared to the old solution where most operators were on all four shifts and also off days were not allocated regularly. From analysis of the results, we can conclude that the initial objectives of reducing overtime and catering for operator preferences have been achieved.

## 6. Conclusion and Future Work

Creating a staff schedule is a challenging and time consuming task. This requires generating a schedule which respects the constraints of the staff and fulfils the organization objectives. Various techniques are used to achieve this such as use of Tabu search algorithms, genetic algorithms linear programming, or mixed integer programming. This paper has proposed a solution for scheduling of IT staff in a bank by using a multiobjective programming method. The solution was implemented using Lingo software and the results were compared with the existing manual method in use. Improvements noted were in reduction in overtime for both the operator and supervisor shift and also the new method allows for the staff to choose the preferred day off. Future work includes implementing the solution in different departments in the live environment in the bank and also adapting the solution to provide flexibility in case of disruptions.

## Figures and Tables

**Table 1:** Results notation.

Number	Identifier	Description
1	M	Morning shift
2	E	Evening shift
3	N	Night shift
4	S	External shift
5	X	Off day

**Table 2:** Supervisors shift—old solution.

	D1	D2	D3	D4	D5	D6	D7	D8	D9	D10	D11	D12	D13	D14	D15	D16	D17	D18	D19	D20	D21	D22	D23	D24	D25	D26	D27	D28	Overtime
Sup1	Vacation													M	M	M	M	M	M	X	N	N	N	N	N	N	X	X	1
Sup2	X	X	E	E	E	E	X	X	M	M	M	M	M	X	X	Vacation													0
Sup3	E	E	M	M	M	M	M	M	N	N	N	N	N	N	N	X	X	X	E	E	E	E	E	X	X	X	M	M	4
Sup4	M	M	X	X	N	N	N	N	N	X	X	E	E	E	E	E	E	E	X	M	M	M	M	M	M	M	N	N	3
Sup5	Vacation																												0

**Table 3:** Supervisors shift—new solution.

	D1	D2	D3	D4	D5	D6	D7	D8	D9	D10	D11	D12	D13	D14	D15	D16	D17	D18	D19	D20	D21	D22	D23	D24	D25	D26	D27	D28	Overtime
Sup1	Vacation													N	N	X	M	E	E	X	M	M	M	M	X	M	M	M	1
Sup2	E	X	M	M	M	M	M	X	M	M	M	M	M	M	E	Vacation													2
Sup3	M	M	X	X	N	N	N	N	N	N	N	N	N	X	M	E	X	M	X	E	N	N	N	X	M	N	X	N	1
Sup4	X	E	E	E	E	E	X	M	N	X	X	E	E	E	X	M	E	X	M	M	E	E	E	N	N	X	N	X	0
Sup5	Vacation																												0

**Table 4:** Operators shift—old solution.

	D1	D2	D3	D4	D5	D6	D7	D8	D9	D10	D11	D12	D13	D14	D15	D16	D17	D18	D19	D20	D21	D22	D23	D24	D25	D26	D27	D28	Overtime
Op1	M	M	M	M	M	E	E	X	N	N	N	N	N	N	N	X	X	X	E	E	E	E	E	X	X	M	M	M	3
Op2	S	S	S	S	S	X	X	E	E	E	X	X	X	N	N	N	N	N	N	X	X	E	E	E	E	E	E	X	1
Op3	N	N	N	N	X	E	E	E	E	E	X	X	X	M	M	N	N	N	N	N	N	N	X	E	E	E	E	E	4
Op4	M	E	E	E	E	X	X	S	S	S	S	S	X	X	M	M	M	M	X	N	N	X	X	E	E	E	E	E	1
Op5	Vacation													E	E	E	E	E	X	X	M	M	M	N	N	N	N	E	2
Op6	X	X	M	M	M	M	M	M	M	M	M	M	M	X	S	S	S	S	S	X	X	N	N	N	N	X	X	X	1
Op7	E	E	E	E	E	X	X	M	M	M	M	M	M	M	X	X	E	E	E	E	E	X	X	M	M	M	M	M	2
Op8	E	M	X	X	N	N	N	N	X	X	E	E	E	E	E	E	X	X	M	M	M	M	M	M	M	X	X	N	0
Op9	N	N	N	N	X	M	M	E	E	E	E	E	E	X	X	M	M	M	M	M	X	S	S	S	S	S	X	X	2

**Table 5:** Operators shift—new solution.

	D1	D2	D3	D4	D5	D6	D7	D8	D9	D10	D11	D12	D13	D14	D15	D16	D17	D18	D19	D20	D21	D22	D23	D24	D25	D26	D27	D28	Overtime
Op1	X	X	M	E	E	E	E	E	E	E	E	E	E	E	N	N	X	E	N	N	X	E	X	N	N	X	E	E	2
Op2	N	N	N	N	N	X	M	N	X	M	M	M	M	X	M	E	E	N	N	X	E	S	S	S	S	S	X	X	2
Op3	E	E	E	N	X	E	X	M	M	M	N	X	X	N	S	S	S	S	S	X	X	M	E	E	E	E	X	M	1
Op4	M	M	M	M	E	X	M	M	M	N	X	X	N	N	X	M	M	M	M	M	M	E	E	X	M	M	M	N	3
Op5	Vacation													M	X	E	N	X	E	E	E	N	X	M	E	E	X	E	0
Op6	S	S	S	S	S	X	X	X	N	X	M	M	M	M	M	M	M	M	M	X	N	X	X	M	M	M	M	M	1
Op7	M	M	X	M	M	N	N	S	S	S	S	S	X	X	E	N	X	N	X	N	N	X	M	E	E	N	N	X	1
Op8	N	N	N	X	M	M	E	E	E	E	E	N	X	X	E	N	N	X	X	M	M	N	N	N	N	X	E	X	1
Op9	E	E	E	E	X	M	E	E	E	E	E	E	E	E	N	X	E	E	E	E	X	M	M	E	X	E	E	E	4
